# Supplementary material for: Biological Role and Clinical Implications of MYOD1L122R Mutation in Rhabdomyosarcoma
Source: Cancers (Basel). 2023 Mar 7;15(6):1644. doi: 10.3390/cancers15061644 (PMC10046495; doi:10.3390/cancers15061644)
Supplement: Supplementary file 1 [file cancers-15-01644-s001.zip › cancers-2192948-supplementary.pdf]

Figure S1. PRISMA 2020 flow diagram for new systematic reviews which included searches of databases and registers only

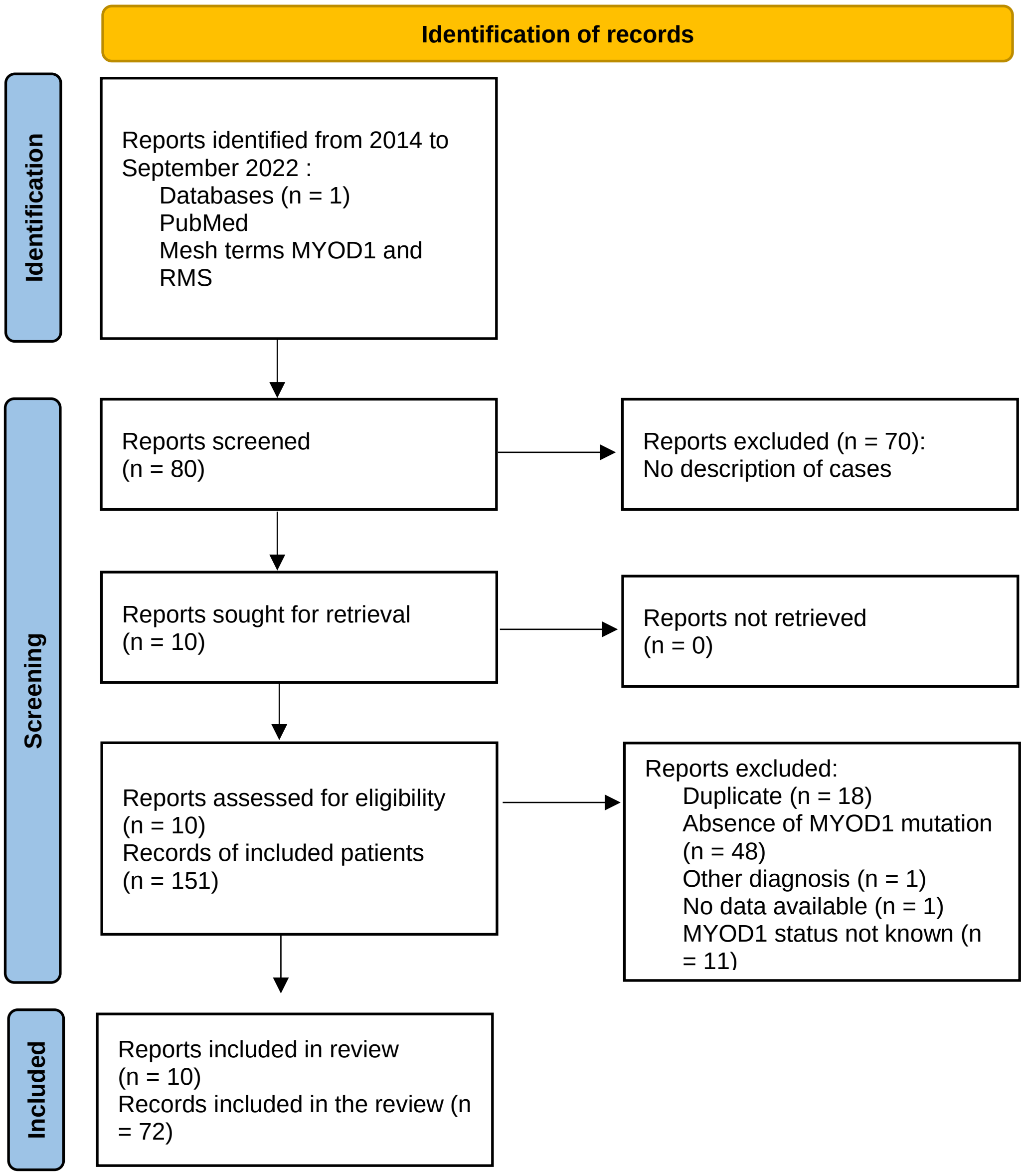

From: Page MJ, McKenzie JE, Bossuyt PM, Boutron I, Hoffmann TC, Mulrow CD, et al. The PRISMA 2020 statement: an updated guideline for reporting systematic reviews. BMJ 2021;372:n71. doi: 10.1136/bmj.n71

For more information, visit: http://www.prisma-statement.org/
